# Treatment planning using MRI data: an analysis of the dose calculation accuracy for different treatment regions

**DOI:** 10.1186/1748-717X-5-62

**Published:** 2010-06-30

**Authors:** Joakim H Jonsson, Magnus G Karlsson, Mikael Karlsson, Tufve Nyholm

**Affiliations:** 1 Department of Radiation Physics, Umeå University Hospital, 90185 Umeå, Sweden; 2 Radiation Physics Section, Department of Radiation Sciences, Umeå University, 90187 Umeå, Sweden; 3 Section of Oncology, Department of Radiation Sciences, Umeå University, 90187 Umeå, Sweden

## Abstract

**Background:**

Because of superior soft tissue contrast, the use of magnetic resonance imaging (MRI) as a complement to computed tomography (CT) in the target definition procedure for radiotherapy is increasing. To keep the workflow simple and cost effective and to reduce patient dose, it is natural to strive for a treatment planning procedure based entirely on MRI. In the present study, we investigate the dose calculation accuracy for different treatment regions when using bulk density assignments on MRI data and compare it to treatment planning that uses CT data.

**Methods:**

MR and CT data were collected retrospectively for 40 patients with prostate, lung, head and neck, or brain cancers. Comparisons were made between calculations on CT data with and without inhomogeneity corrections and on MRI or CT data with bulk density assignments. The bulk densities were assigned using manual segmentation of tissue, bone, lung, and air cavities.

**Results:**

The deviations between calculations on CT data with inhomogeneity correction and on bulk density assigned MR data were small. The maximum difference in the number of monitor units required to reach the prescribed dose was 1.6%. This result also includes effects of possible geometrical distortions.

**Conclusions:**

The dose calculation accuracy at the investigated treatment sites is not significantly compromised when using MRI data when adequate bulk density assignments are made. With respect to treatment planning, MRI can replace CT in all steps of the treatment workflow, reducing the radiation exposure to the patient, removing any systematic registration errors that may occur when combining MR and CT, and decreasing time and cost for the extra CT investigation.

## Background

Computed tomography (CT) has been the basis for treatment planning since the introduction of 3D conformal radiotherapy because of its availability, high geometrical accuracy, and direct connection to electron density used in dose calculations. From the beginning, however, it has been clear that CT alone does not always provide enough information for an accurate delineation of the target volume. Magnetic resonance (MR) imaging adds significant value in delineations of prostate targets [1-3], brain lesions [4,5], and head and neck tumors [6]. In addition, a recent report notes that MR may help distinguish lung tumors from surrounding atelectasis [7]. Although clinics now use multimodality imaging as a basis for target delineation, CT is still the preferred choice for treatment planning. The use of CT for treatment planning, however, is not unproblematic. The extra costs associated with multiple imaging modalities have motivated several groups to study the possibility of developing treatment plans using only MR images [8-10]. Other groups refer to the additional uncertainty introduced with the registrations between CT and MR as a motivation for treatment planning that directly uses MR images [10-13]. Errors introduced in the registration will affect the treatment systematically throughout the entire treatment period. Prostate and gynecological patients are especially problematic as the patients can have different rectal and bladder filling during the different imaging sessions. This implies that the registration result can significantly depend on the surrounding tissues and in itself introduce significant uncertainty [14,15]. The geometrical distortions and the lack of electron density information are the main obstacles associated with using MR images when developing treatment plans.

Geometrical distortions are caused by nonlinearities in the magnetic gradients, inhomogeneities in the static magnetic field, and chemical shift or magnetic susceptibility artifacts. In modern MR scanners, the problems with field inhomogeneities are limited and the strong gradients have increased the problems with gradient nonlinearities [16]. Nonlinearities can be characterized and corrected using spherical harmonics expansions of the fields generated by the gradient coils [17]. These algorithms have proved successful [18] and provide adequate geometrical accuracy for radiotherapy purposes and are now implemented as a standard clinical tool in the Siemens MR software (ver. B15). Chemical shift artifacts and distortions induced by magnetic susceptibility variations have been investigated with a focus on prostate treatments and the effect is shown to be small for internal structures relevant for prostate treatments [10]. In general, modern sequences such as 3D turbo spin echo sequences using relatively high bandwidth reduce distortions caused by susceptibility differences in tissue/bone and air/tissue interfaces to an acceptable level for radiotherapy.

Modern treatment planning systems often use a conversion of the Hounsfield numbers to relative electron density to calculate doses. This can be done through use of generic formulas [19] or via a tissue look up table. A look up table can help account for the effects of variations in atomic number Z between different tissues, a technique that can provide more detailed information about the cross sections for different interactions. This can make a significant difference for calculations on proton or ion beams [20], whereas photon beam calculations are rather insensitive to uncertainties in the electron density [21]. There is no relation between MR image values and electron density as is the case for CT. One possible way to overcome this is to ignore the variations in electron density in the patient, i.e., turn off the inhomogeneity correction. Using this approach to calculate doses, several groups have noted dose differences ranging from 0.9% to 2.5% [10,22,23]. For brain treatments, the difference in dose with and without inhomogeneity correction has been reported to be in the range 1-1.5% [13,24]. Segmentation and bulk density assignment of relevant tissues can improve accuracy. For prostate patients, the average difference in mean dose to target compared to CT calculations has been reported to be lower than 0.5% with the bulk density approach [10,23]. For brain tumors, Kristensen et al. report mean deviations of the same magnitude [24].

In this study, we verify results from previous work in the pelvic area and brain and further investigate the dose calculation accuracy for bulk density assigned geometries (synthetic CT) in both the thoracic and the head and neck regions. We also aim to find the most suitable bulk densities for pelvic bone, skull bone, and pulmonary tissue. Finally, we aim to decide whether or not the dose calculation accuracy for bulk density assigned MR is sufficient for clinical radiotherapy treatment planning in all investigated areas.

## Methods

### Subjects

In this retrospective study, we analyzed imaging data from patients in four different anatomical regions: prostate (n = 10), thorax (n = 10), brain (n = 10), and head and neck (n = 10). The patients included in each subgroup were randomly selected. Table 1 lists patients and data.

**Table 1:** Patient population

Anatomic region	Female	Male	Mean age (range)	Mean number of fields
Prostate	-	10	67.0 (62-74)	4.3
Brain	1	9	69.1 (42-80)	4.2
Thorax	4	6	64.6 (46-85)	3.3
Head & Neck	3	7	66.5 (41-81)	4.0

### Imaging

No images were acquired solely for this study because imaging with both CT and MR are part of the standard clinical routine in our department. Prostate and thoracic patients were imaged in treatment position with the MR scanner (Espree 1.5 T, Siemens, Erlangen, Germany) using standard fixation equipment. This was not possible for the head and neck and brain patients as the fixation devices were not compatible with the head and neck coils. A T2 weighted turbo spin echo 3D sequence (matrix size - 384 × 384, slice thickness - 1.7 mm, TR - 1500 ms, TE - 209 ms, bandwidth - 592 Hz per pixel) covering the patient outline in the treatment area was used for the prostate patients. The thorax patients were imaged with a half Fourier turbo spin echo-sequence (matrix size - 320 × 320, slice thickness - 5 mm, TR - 579 ms, TE - 53 ms). A pace navigator was used to reduce the motion artifacts from breathing. The images were corrected for geometrical distortions introduced by nonlinearities in the gradients using the standard Siemens 3D distortion correction algorithm. A flat bed insert and a standard radiotherapy mattress were placed on the spine coil to create similar bed stiffness and shape as the radiotherapy couch.

For all examinations, the CT imaging was performed with a GE Lightspeed scanner (GE Medical Systems, Milwaukee, Il, USA) equipped with a carbon fiber radiotherapy couch (Siemens, Erlangen, Germany) with slice thickness 2.5 mm and 130 kV. The CT scanner HU-scale is calibrated regularly using a standard phantom provided by the vendor for each available CT tube voltage. The HU homogeneity was verified using a CATPHAN 600 phantom (The Phantom Laboratory, Salem, NY, USA), and the peripheral HU value varied less than 4 HU (0.4% of the attenuation coefficient of water) compared to the HU value in the center of the phantom.

### Structure definition and treatment plans

The patients selected for this retrospective study had all been previously treated and had complete clinical treatment plans with targets defined by experienced physicians and treatment plans constructed by radiotherapy assistants based on the CT study. The MR and CT studies had all been previously registered; at our department, the target volume is defined on MR images registered to the CT study. All plans were three-dimensional conformal treatments. Oncentra Masterplan (Nucletron B.V., Veenendaal, Holland) was used for all delineations, registrations, and dose calculations.

**Figure 1:**
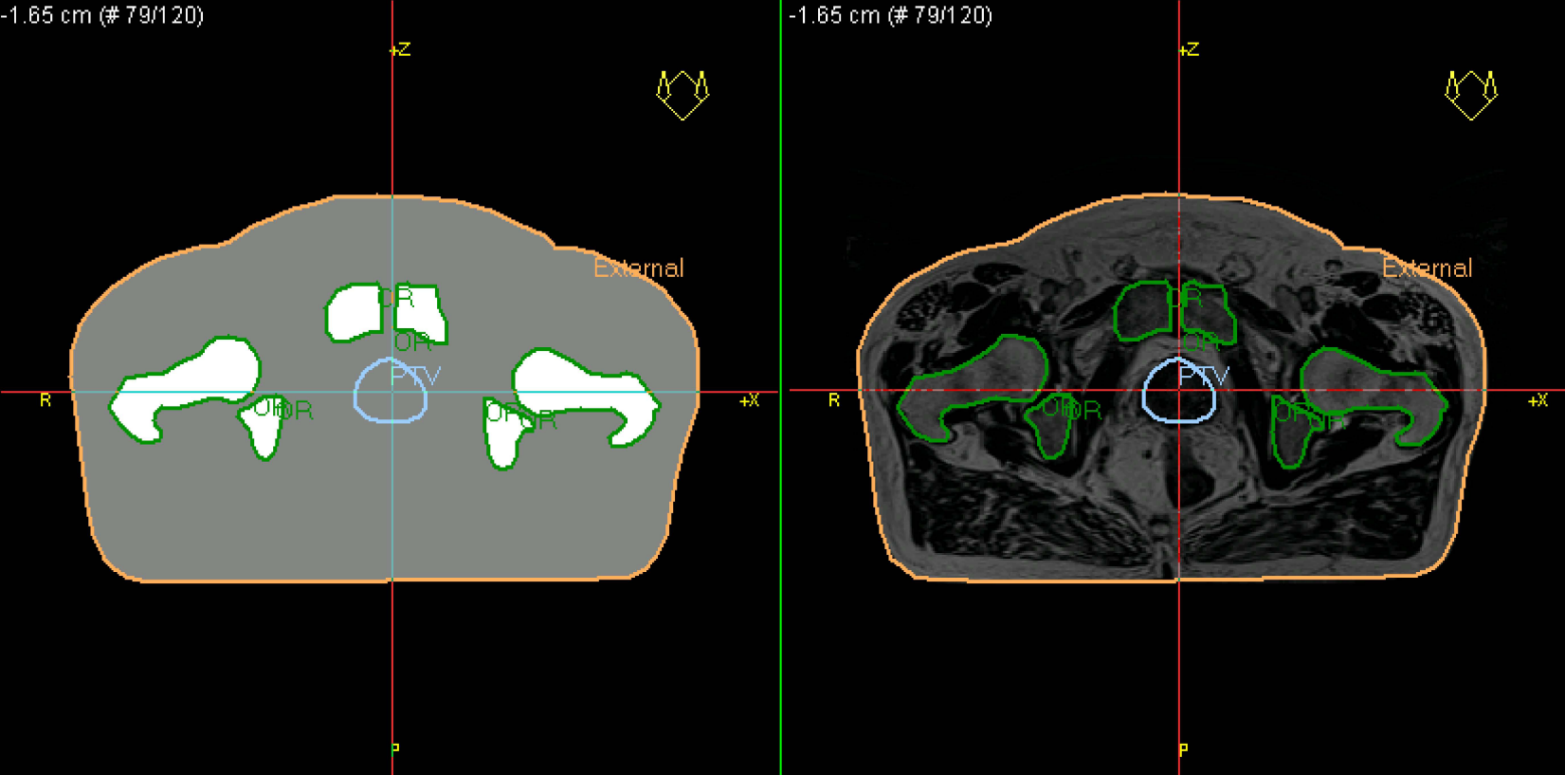
**Synthetic CT and MR image**. The synthetic CT with assigned mass densities (left) and the MR image on which it was based (right).

For both the CT and MR studies, we manually delineated the additional structures needed for comparison of bulk density treatment plans and the clinical CT-based treatment plans. For prostate patients, this included the patient outline, femur, femoral head, and hipbone; for thorax patients, this included the lung. Since the head and neck and brain patients were not imaged in treatment position in the MR scanner, the bulk density structures were delineated only on the CT images for those patients, i.e., the patient outline, skull bone, and air cavities. Bone was considered as one tissue type: the cortical and the trabecular parts were delineated together. The different structures were assigned mass densities to form a synthetic CT image (Figure 1). The treatment planning system uses a look up table to map the mass densities to electron densities used for dose calculations. The target volumes that were used in the clinical treatments were used for all dose calculations.

### Dose calculations

The dose calculations were performed using the same field setup in four geometries: 1.) the CT geometry with heterogeneity correction (the normal clinical geometry); 2.) no heterogeneity correction on CT data (the patient external contour delineated on CT and the entire patient anatomy set to water); and 3.) bulk density geometry based on CT data for all treatment regions and 4.) bulk density geometry based on MR data for the prostate and thorax regions. The tumor volumes were all delineated on MR. The mass densities, as recommended in ICRU 46 [25], are cranium (whole) - 1.61 g/cm^3^, femoral bone (whole) - 1.33 g/cm^3^, lung - 0.26 g/cm^3^, and average soft tissue 1.025 g/cm^3^. Air was set to 0.001 g/cm^3^. In all cases, mass density values correspond to healthy adults. For soft tissue, the mean value for female and male is given. The collapsed cone calculation algorithm was used for the lung patients, while the pencil beam algorithm was used for all other calculations (following the normal clinical procedures at our department).

### Evaluation

The study was divided into two steps: (i) evaluation of the shape differences of the dose volume histograms (DVHs) for the different calculation geometries using the clinical treatment plan and (ii) comparison of the number of monitor units (MUs) required to reach the prescribed dose with the different calculation geometries using the clinical beam setup.

The DVH for the target from the CT calculation was compared with the DVH for the bulk densities recommended by the ICRU for bone and lung and with the exact same treatment plan, i.e., the same beam setup and number of MU per beam. In this part of the study, we investigated what impact the bulk density approach had on the DVH shape and assessed the sensitivity of the DVH to the bulk density assignment. Bulk densities for DVH assessment were defined on CT geometry.

In the second part, the total number of MUs required to reach the prescribed dose was used to quantify the impact of the different calculation geometries. This approach is almost equivalent with the method of comparing the dose for a fixed number of MUs [11,23,24], but we see it as more intuitive since it is the number of MUs rather than the prescribed dose that will be affected by the change in calculation geometry. All treatment plans were normalized with respect to the mean dose in the primary target volume (PTV). Because the different beams for each plan were energy fluence weighted, the MU relation between the beams were independent of the calculation geometry.

## Results

### Evaluation of DVH

**Figure 2:**
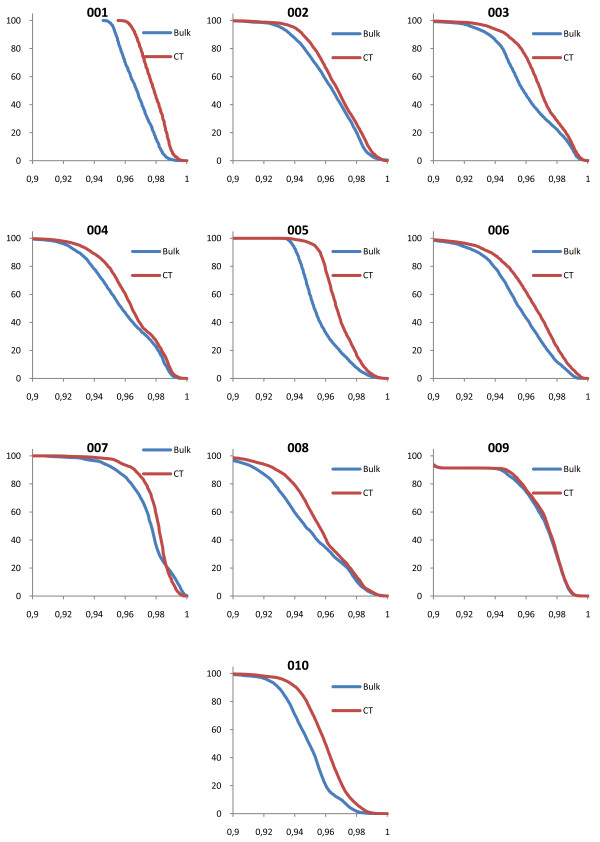
**DVH comparisons for all geometries**. The figure shows PTV DVH comparisons between bulk density assigned data and CT data for the prostate patients. The exact same treatment setup was used for the two geometries, including number of MUs given. The DVHs have been normalized to the maximum dose from the CT DVH.

**Figure 3:**
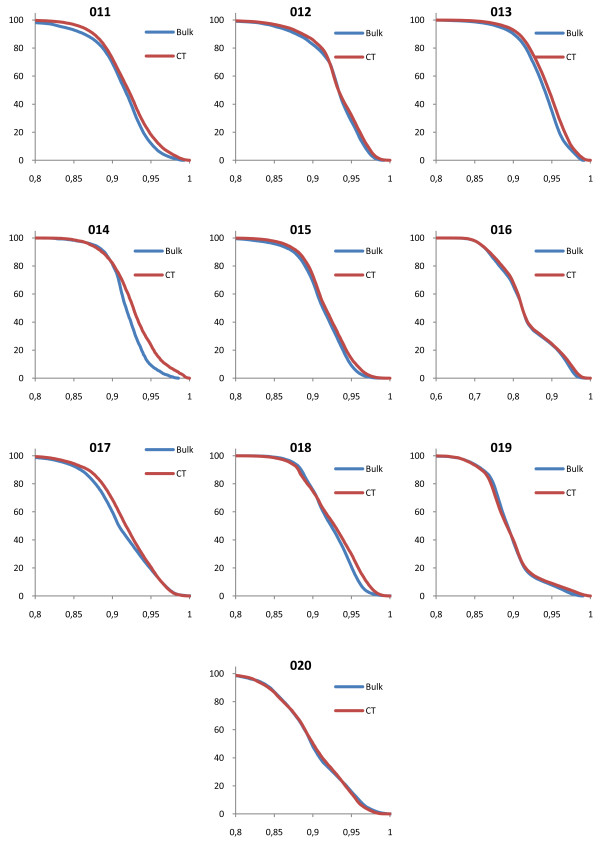
**DVH comparisons for all geometries**. The figure shows PTV DVH comparisons between bulk density assigned data and CT data for the lung patients. The exact same treatment setup was used for the two geometries, including number of MUs given. The DVHs have been normalized to the maximum dose from the CT DVH.

**Figure 4:**
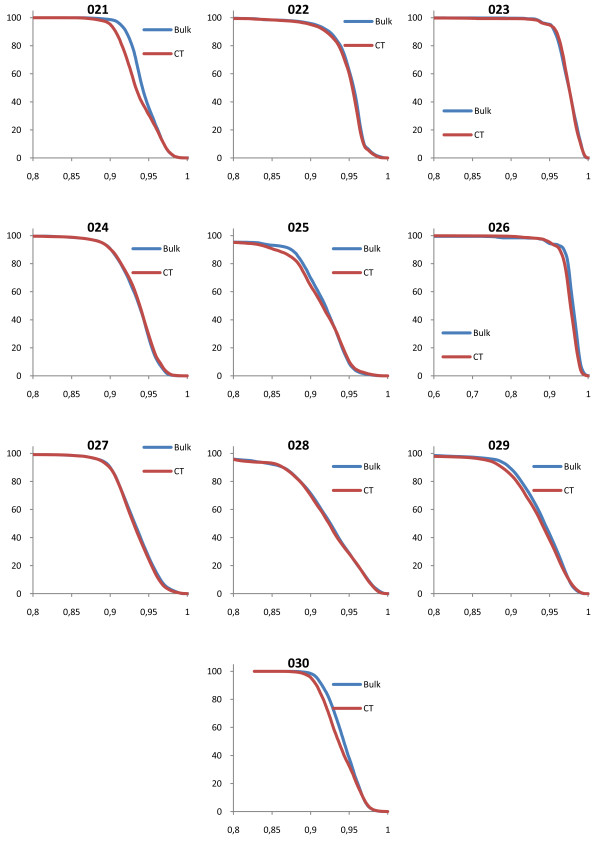
**DVH comparisons for all geometries**. The figure shows PTV DVH comparisons between bulk density assigned data and CT data for the head and neck patients. The exact same treatment setup was used for the two geometries, including number of MUs given. The DVHs have been normalized to the maximum dose from the CT DVH.

**Figure 5:**
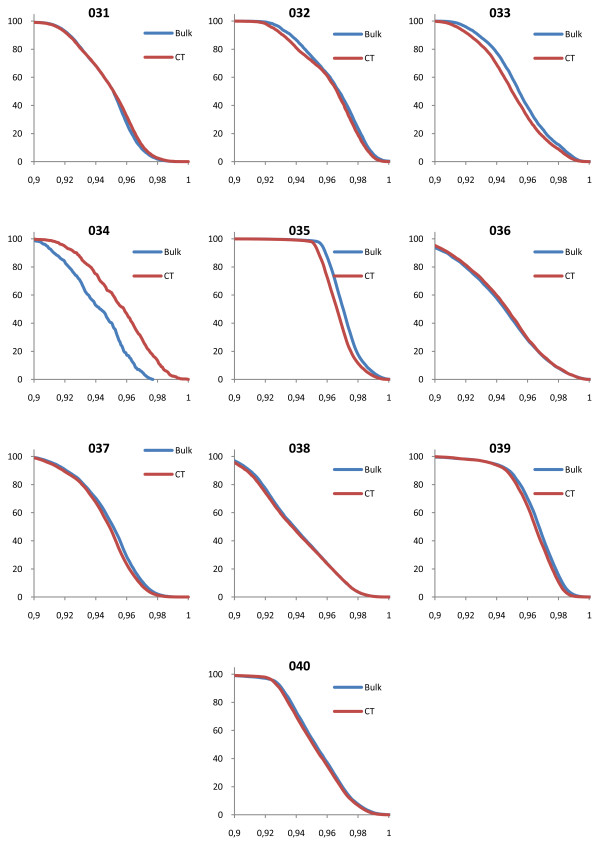
**DVH comparisons for all geometries**. The figure shows PTV DVH comparisons between bulk density assigned data and CT data for the brain patients. The exact same treatment setup was used for the two geometries, including number of MUs given. The DVHs have been normalized to the maximum dose from the CT DVH.

The shapes of the target volume DVHs were fairly insensitive to the bulk density assignment Figure 2, figure 3, figure 4, figure 5 and table 2 also show that the density values recommended in ICRU 46 [25] provide a clinically acceptable agreement between bulk density DVH and DVH based on the CT study. Therefore, we used these relative mass densities in the second part of the study where the number of MUs required to reach the prescribed dose was evaluated.

**Table 2:** Quantitative DVH information

	**Mean difference CT**_**bulk **_**- CT**	
Treatment	D95 [range] %	D50 [range] %
Prostate	-0.96 [-1.44; -0.21]	-0.82 [-1.44; -0.08]
Thorax	-0.56 [-2.47; 0.46]	-0.36 [-0.93; 0.15]
Brain	0.07 [-1.14; 0.60]	-0.01 [-1.51; 0.42]
Head & Neck	0.68 [-0.50; 2.17]	0.27 [-0.21; 0.80]

D95 and D50 (dose covering 95% and 50% of the ROI respectively) differences in mean value between the CT based calculations and bulk density calculations based on CT geometry, given in percent of maximum dose in the CT calculations.

### Evaluation of dose calculations

Table 3 lists the mean values and standard deviations of the relative differences in MUs between the different calculation geometries and the standard CT geometry. The mean MU values of the bulk density assigned plans were within 1% of the CT plans for all patient groups. There was a consistent improvement of the calculation accuracy with bulk density assignment compared to calculations performed without inhomogeneity corrections, except in the head and neck plans where bulk density assignment gave the same result.

**Table 3:** Calculation geometry comparisons

	MR_bulk_/CT		CT_bulk_/CT		CT_hom_/CT	
Treatment area	Mean [range] %	St.d. %	Mean [range] %	St.d. %	Mean [range] %	St.d. %
Prostate	0.2 [-0.8; 0.9]	0.5	0.8 [0.1; 1.1]	0.3	-1.6 [-2.3; -1.6]	0.2
Thorax	0.2 [-0.6; 0.9]	0.4	0.5 [0.0; 1.0]	0.3	1.4 [-0.8; -6.5]	2.1
Head&Neck	-	-	-0.3 [-0.8; 0.1]	0.3	-0.3 [-1.1; 0.6]	0.5
Brain	-	-	0.0 [-0.7; 1.5]	0.6	-1.5 [-2.4; -0.7]	0.5

The table shows comparisons between the different calculation geometries and normal CT geometry in percent. **MR**_bulk _designates bulk density assigned MR data. **CT**_bulk _designates bulk density assigned CT data. CThom designates calculations performed without inhomogeneity corrections on CT data.

## Discussion

**Figure 6:**
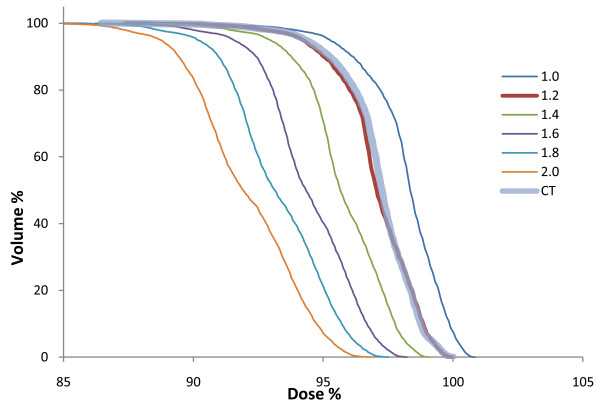
**DVH for prostate PTV for several bone bulk densities**. PTV DVH comparison for several bulk density assignments of femoral bone to CT geometry. The treatment setup and MUs given are the same in all cases. The DVHs are normalized to the CT maximum dose.

In general, the shape differences, D95 and D50 between PTV DVHs based on full CT data compared to bulk density data were small; however, in the prostate patients there is a clear underdosage when the bone bulk density recommended for healthy adults (1.330 g/cm^3 ^according to the ICRU) was used. Figure 6 - a single prostate patient DVH plotted for multiple bone bulk densities - shows that there is evidence that a lower bulk density value closer to 1.2 g/cm^3 ^would give results closer to the CT calculation. The value recommended by the ICRU for 90 year-old adults is 1.220 g/cm^3^. It also appears that rather drastic variations in the assigned relative density give only a modest change of the calculated dose. The geometry that was most sensitive to the choice of bulk density value in the present study is the prostate case where the femoral head and the pelvic bone effects the radiation field, but even in this case a variation in relative mass density from 1.2 g/cm^3 ^to 1.4 g/cm^3^, an increase of 15%, changes the dose by only 1-2%.

The differences in dose calculation results when based on CT and bulk densities are small (Table 3). The largest observed deviation in MUs for an individual patient after bulk density assignments was 1.6%. This should be seen in the light of the uncertainty of the total standard calculation, which has been estimated to 3.2% in ICRP publication 86 [26]. Adding these values in quadrature yields a total cumulative error of 3.6%, which is a noticeable increase. However, the benefit of increased geometrical accuracy by eliminating the image registration step between the MR and CT dataset in the treatment planning [14] should be weighed against the small increase in dose calculation uncertainty.

**Figure 7:**
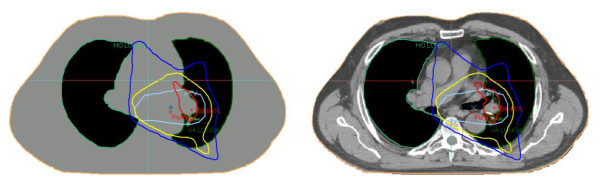
**Dose distribution in lung for synthetic and normal CT**. The dose distribution in the thoracic area in a bulk density based-treatment plan (left) and in a CT-based treatment plan (right). The PTV is light blue, the 70% isodose is blue, 95% is yellow, and 105% is red.

The thorax patients that were investigated in this study showed very good agreement between CT and the bulk density approach, considering the difficult geometries at these sites. The ribcage was not segmented because of the very troublesome and time-consuming task of manual segmentation and because the effect on the radiation beam caused by the bone should be minor compared to the impact of lung tissue. As seen in figure 7, the distortions in the dose distributions are relatively small even in this inhomogeneous PTV that includes pulmonary tissue and air gaps.

The head and neck cases that were investigated were uncomplicated from a radiotherapy point of view since IMRT treatments were excluded from the study. However, the treatment plans that were investigated yielded good results and suggest that the use of MR-based synthetic CT may be used to decrease the impact of dental filling artifacts in head and neck cases. The bulk density approach on head and neck cases has been successfully used [27] when applied to CT images.

Even though differences in imaging setup prohibited study of bulk density images based on MRI in head and neck and brain, the validity of the bulk density approach is established by assigning bulk densities to the CT images. There is no reason to suspect that the accuracy would be significantly altered by delineating the bulk density geometries on MR images.

**Figure 8:**
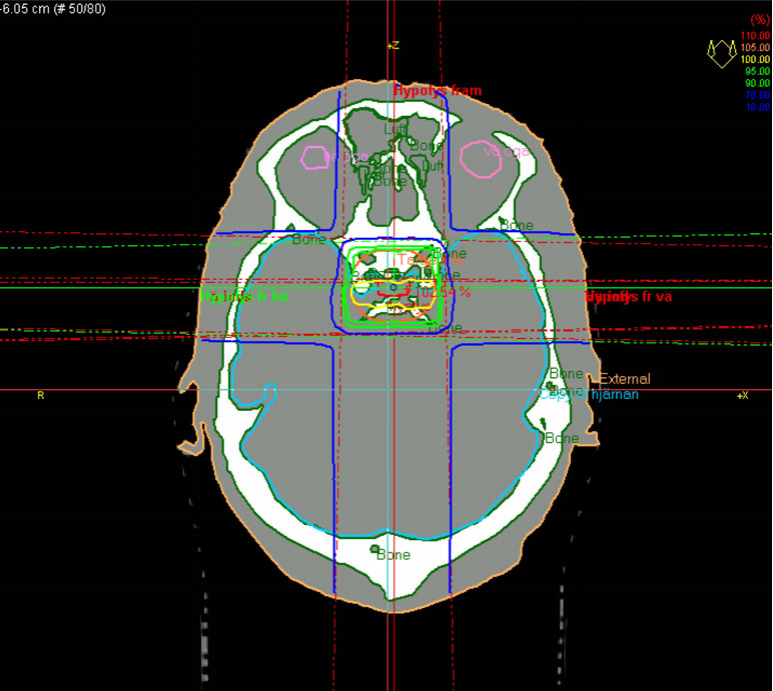
**An inhomogeneous PTV**. A particularly difficult case where the PTV is very small and inhomogenous, leading to a larger than normal deviation of bulk density dose calculation compared to CT calculation.

Except for the prostate cancer cases where there is a systematic difference between synthetic CT and normal CT calculations, patient number 034 had the worst corrsepondence between the DVH based on CT and the DVH based on bulk density assingments (figure 5). For this patient, the PTV was very small and inhomogeneous, located in the hypothalamus area of the brain (figure 8), which makes the case challangeing from a dose calcualtion perspective. Despite the difficult geometry, the difference in MUs was only 1.5%.

Geometrical distortion is a known problem connected to MR in radiotherapy [16,17]. In modern scanners, patient-independent distortions are mainly due to nonlinearities in the gradients and to minor part due to inhomogeneities in the static magnetic field B_0_. Gradient nonlinearities are a direct consequence of the gradient coil design and can be described and corrected using generic methods. In the present study, a 3D correction algorithm based on a spherical harmonic expansion of the fields generated by the gradient coils was used [17]. Siemens guaranties a B_0 _homogeneity of < 4 ppm within an elliptical field of view with axis 45 × 45 × 30 cm^3^. For a sequence with bandwidth 592 Hz/pixel (as used in the current study and with a 1.5 T scanner), this corresponds to a distortion of less than 0.5 pixels. Magnetic susceptibility induced distortions as well as B_0 _inhomogeneity-related distortions can be minimized using a high bandwidth sequence. In extreme situations, the susceptibility effects close to air/water interfaces can reach 10 ppm [28]. This corresponds to a distortion of around 1 pixel for the sequence used in the present study. Generally, dose calculations for photons are insensitive to small geometrical errors. Quality control of the geometrical distortions is important, however, when it comes to target definition and patient positioning. The present study shows that from a dose calculation perspective MR planning is feasible. Detailed broader analyses are needed before clinical implementation.

In the present work, we have only dealt with conformal 3D treatments. For this purpose, we deemed that a comparison of the calculated number of MUs needed to reach the prescribed dose was an adequate quality measure. However, if the same study should be performed for patients treated with IMRT, a different methodology should be used so that the dose distributions can be compared in voxel-wise fashion. With IMRT, the calculated dose distribution is used as feedback in an iterative optimization process. This means that there is a risk for increased sensitivity to small errors in the anatomy segmentation used for the bulk density assignment.

A large-scale implementation of treatment planning on MR data relies on effective methods for delineation of structures and bulk density assignments. Automatic segmentation of bone-e.g., by using deformable atlas-to-patient image registration [29]-eliminates the need for manual segmentation and improves the efficiency of the workflow. In addition, the MR coils for the head and neck area must be revised to accommodate the fixation devices that keep the patient immobilized during treatment so that the plan can be constructed in the correct geometry. MR coils that are compatible with these fixation devices are being constructed at our department in collaboration with Umeå Institute of Design.

## Conclusions

We conclude that the dose calculation accuracy is not a limiting factor for radiotherapy treatment planning solely using MR images when using a bulk density approach, even in the case of tissues that differ largely from water such as pulmonary tissue. The density values that are recommended by the ICRU yield accurate results. In the prostate patients, the femoral bone density should be 1.220 g/cm^3 ^as recommended by the ICRU for 90 year-old patients. Treatment planning using MR images makes the CT unnecessary in the radiotherapy workflow. Using only MR images reduces the radiation exposure to the patient, removes any systematic registration errors that may occur when combining MR and CT, and eliminates the time and cost associated with the extra CT investigation.

## Competing interests

The authors declare that they have no competing interests.

## Authors' contributions

JJ performed the dose calculations and drafted the manuscript. TN conceived the study and participated in its design and helped draft the manuscript. MGK participated in the design of the study and gathered all data. MK participated in the design and coordination of the study. All authors read and approved the final manuscript.
